# Structure of Rhomboid Protease in a Lipid Environment

**DOI:** 10.1016/j.jmb.2011.01.029

**Published:** 2011-03-25

**Authors:** Kutti R. Vinothkumar

**Affiliations:** MRC Laboratory of Molecular Biology, Hills Road, Cambridge CB2 0QH, UK

**Keywords:** membrane proteins, intramembrane protease, rhomboid, lipid bilayer, X-ray and electron crystallography, TM, transmembrane, DMPC, dimyristoyl phosphatidylcholine, CHAPSO, 3-[(3-cholamidopropyl) dimethylammonio]-2-hydroxy-1-propanesulfonate, 3D, three-dimensional, 2D, two-dimensional, Aqp0, aquaporin, WT, wild-type, PDB, Protein Data Bank, DM, n-Decyl-β-D-maltopyranoside

## Abstract

Structures of the prokaryotic homologue of rhomboid proteases reveal a core of six transmembrane helices, with the active-site residues residing in a hydrophilic cavity. The native environment of rhomboid protease is a lipid bilayer, yet all the structures determined thus far are in a nonnative detergent environment. There remains a possibility of structural artefacts arising from the use of detergents. In an attempt to address the effect of detergents on the structure of rhomboid protease, crystals of GlpG, an *Escherichia coli* rhomboid protease in a lipid environment, were obtained using two alternative approaches. The structure of GlpG refined to 1. 7-Å resolution was obtained from crystals grown in the presence of lipid bicelles. This structure reveals well-ordered and partly ordered lipid molecules forming an annulus around the protein. Lipid molecules adapt to the surface features of protein and arrange such that they match the hydrophobic thickness of GlpG. Virtually identical two-dimensional crystals were also obtained after detergent removal by dialysis. A comparison of an equivalent structure determined in a completely delipidated detergent environment provides insights on how detergent substitutes for lipid. A detergent molecule is also observed close to the active site, helping to postulate a model for substrate binding and hydrolysis in rhomboids.

## Introduction

Membranes define the external boundary and internal compartments in a cell. Proteins in such membranes are dynamic and perform a wide variety of functions including transport, energy transduction, and signalling. Membrane proteins are amphiphilic, and thus their study often involves extraction from the bilayer using detergents. Although detergents mimic many properties of lipids, they can disrupt the structure and activity of membrane proteins.^1–3^ Lipid molecules are frequently lost during purification, being replaced by an annulus of detergent. However, in many instances, lipids have been observed in crystal structures binding to specific sites, and often these lipid molecules are essential for the activity of proteins.^4–7^ Only in a handful of membrane protein structures are ordered lipid molecules observed surrounding the entirety of the protein to form an annulus. These include bacteriorhodopsin^8,9^ and aquaporin (Aqp0),^10,11^ which form ordered two-dimensional (2D) lipid-containing arrays, the central cavity of the oligomeric ring of subunit K of V-ATPase,^12^ and a potassium channel^13^ where lipids were added during purification. It is a desirable goal to obtain structures of membrane proteins in a native lipid environment to help understand their function, to show how they interact with lipids, and to validate the role and limits of the use of detergents, which provide an artificial stabilising environment for membrane proteins.

Intramembrane proteases are one class of membrane proteins, which share the property of cleaving transmembrane (TM) proteins to release cytoplasmic, luminal, or extracellular domains that move to new locations where they can carry out their biological functions.^14,15^ The intramembrane protease family includes serine, aspartyl, and metallo-proteases. All have multiple TM helices with active-site residues sequestered within distinct TM domains, and all carry out peptide bond hydrolysis within the bilayer.^16^ Rhomboids, first discovered as part of the epidermal growth factor signalling pathway in *Drosophila*,^17^ are characterised by a core of six TM helices.^18^ Structures of the rhomboid protease GlpG from *Escherichia coli* and *Haemophilus influenzae* have revealed that the catalytic dyad serine and histidine forms part of a water-filled cavity separated from the external medium by a flexible loop and from the bilayer by TM helices.^19–22^ Substrate proteins are thought to diffuse laterally through gaps between TM helices to reach the rhomboid active site. In support of this idea, multiple structures of rhomboids show large variations in one of the TM helices whose movement has been predicted to be essential to allow substrate entry and, subsequently, catalysis.^20,21,23^ Biochemical studies, molecular dynamics simulation, and the structure of GlpG in complex with a covalently bound inhibitor provide additional indications of structural change in the protein.^24–26^ Since all these structures have been determined in a detergent environment, it is unclear whether such structural changes are required and whether the presence of detergent has affected the native state of rhomboids. As a crucial step in understanding the mechanism of these very interesting enzymes, I describe the structure of rhomboid protease in a lipid environment, its comparison with the structure of a completely delipidated protein in a detergent environment, and a model for substrate binding and catalysis.

## Results

### Structures of GlpG S201T

**Fig. 1:**
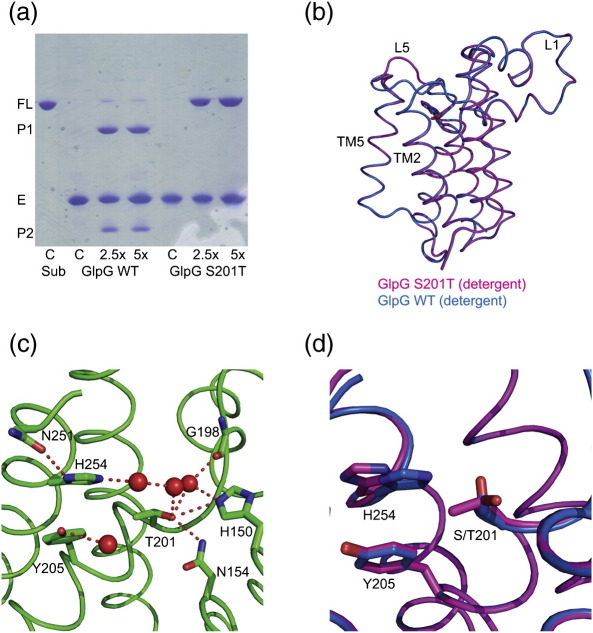
An active-site mutant of GlpG. (a) A rhomboid activity assay showing the cleavage of an artificial substrate (LacY TM2) expressed as a fusion protein to maltose-binding protein and thioredoxin. The cleavage at a specific site in LacY TM2 of the full-length (FL) substrate results in two products. The product (P1) with MBP migrates at ∼ 55 kDa, and a second product (P2) with thioredoxin migrates at ∼ 24 kDa. The mutation of serine to threonine at the active site results in an inactive enzyme with no cleavage of the substrate. Lanes marked C denote the control for substrate (sub) or enzyme (E). Substrate was used at a concentration of ∼ 2 μM, and enzymes were used at concentrations of 5 or 10 μM. A large excess of enzyme was used to test whether the S201T mutant had any residual activity. (b) An overlay of the structures of GlpG WT and S201T, determined in the trigonal crystal form in a detergent environment. The rmsd for the C^α^ atoms between these structures excluding L5 (residues 245–249) is 0.15 Å. (c) The active site of GlpG S201T: Replacement of active-site serine by threonine results in the displacement of the active-site histidine, such that it is within hydrogen-bonding distance of the side chain of Asn251. In the WT structure, a water molecule mediates this interaction. The hydroxyl of Thr201 points towards the oxyanion hole, which is characterised by the main-chain amides of residues Leu200 and Ser201, the imidazole nitrogen of His150, and the side-chain amide of Asn154. These residues are thought to stabilise the negative charge on the carbonyl oxygen formed during proteolysis. The hydroxyl of Thr201 is within hydrogen-bonding distance to the side chains of His150 (3.3 Å) and Asn154 (3.12 Å). This and the fact that it points away from the active-site histidine explain why this mutant is inactive. (d) The overlay of the active sites of WT and S201T shows clearly the extent of histidine side-chain movement.

There were two potential reasons for wishing to observe the effect of mutating the active-site serine to threonine. First, the hydroxyl of threonine might simply substitute for the serine hydroxyl to retain the activity, or second, if the threonine mutant were inactive, it would be a good tool to trap enzyme–substrate complexes. In practice, this mutant was inactive (Fig. 1a). Two different crystal forms of GlpG S201T were obtained. The first one was obtained from a completely delipidated protein as described previously for the wild-type (WT) protein^19,26^ in a trigonal crystal form (space group, *R*32) and diffracted to 1.85 Å (Table 1). The structures of GlpG WT and S201T in this crystal form are largely identical (Fig. 1b) except for a small difference in L5 (rmsd for C^α^ atoms excluding L5 is 0.15 Å). The hydroxyl of Thr201 points away from active-site His254 and is within hydrogen-bonding distance to the side-chain amide of Asn154 and the imidazole nitrogen of His150, both residues being part of the oxyanion hole,^26^ thus explaining the inactivity (Fig. 1c). The mutation of serine to threonine results in a displacement of side chain of the active-site histidine by ∼ 1 Å (Fig. 1d). Apart from a small displacement of the active-site histidine, this mutation preserves the geometry of the active site of GlpG. Even the network of hydrogen bonds mediated by water molecules remains intact (Fig. 1c).

**Fig. 2:**
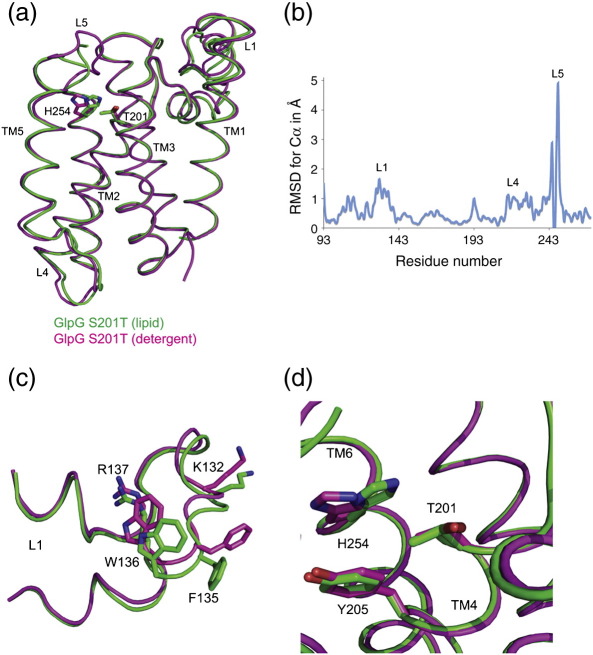
Comparison of GlpG S201T structures in lipid and detergent environments. (a) An overlay of the two structures and (b) a graphical representation of the rmsd, calculated with Superpose in CCP4^28^ suite, show the differences in the loop regions, particularly L1, L4 and L5. The graphical plot shows the main-chain rmsd along the length of the polypeptide. The average rmsd deviation is 0.664 Å for the C^α^ atoms. (c) A minor rearrangement in L1 as a result of crystal contact and to accommodate the lipids, which includes changes in the rotamer conformations of Lys132 and Phe135. (d) In the active site, the side chain of His254 shows a more prominent displacement in the GlpG S201T structure in a detergent environment.

The second crystal form of GlpG S201T was obtained by using a lipid detergent mixture, dimyristoyl phosphatidylcholine (DMPC)/3-[(3-cholamidopropyl) dimethylammonio]-2-hydroxy-1-propanesulfonate (CHAPSO), which has a tendency to form bicelles or bilayers.^27^ These crystals belonged to the orthorhombic space group *P*2_1_2_1_2_1_ and diffracted to 1.7 Å (Table 1). A comparison of this structure obtained in a lipid environment with the GlpG S201T in a detergent environment shows major differences only in the loop regions (Fig. 2a and b), which are reflected in their temperature factors (Supplementary Fig. S1). Changes observed in L1 are minor, predominantly as a result of crystal contacts and to accommodate the lipid molecules (Fig. 2b). The displacement of His254 due to S201T substitution is much more pronounced in the structure determined in a detergent environment than in the structure determined in a lipid environment. The exact reason for this is not clear (Fig. 2c). An elongated density, which could accommodate ∼ 4 carbon atoms, is observed just above the active-site threonine in the structure of GlpG S201T determined in a lipid environment (Supplementary Fig. S2). It is unclear what this density represents, as none of the components used in crystallisation can explain the density exactly. Hence, it was not modelled in the present structure.

### An asymmetric bilayer around GlpG

**Fig. 3:**
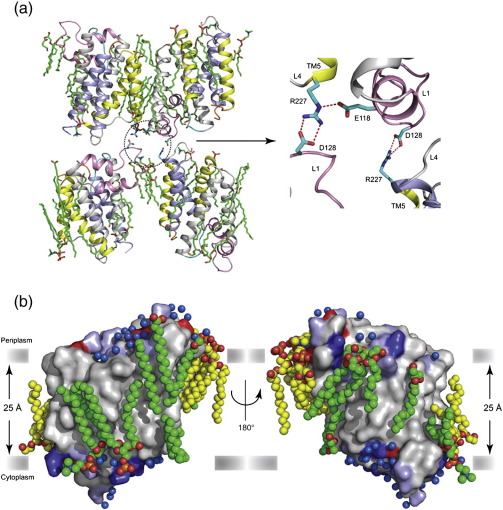
Structure of GlpG in a lipid environment. (a) Type I crystals of GlpG showing an alternating up and down orientation of GlpG molecules (shown in cartoon representation) in the same layer with a shift along the *a*-axis. Within each layer, lipid molecules (green sticks) along with TM1 and TM5 mediate the lateral interaction between protein molecules. Two membranous layers are held together by interactions (marked with dotted circle) between residues of symmetry-related molecules in L1 and L4. The polar contacts mediated by side chains of charged residues (shown in stick representation) play a major role in this interaction. The interaction between E118, D128, and R227 is shown separately as a magnified view with the lipids removed. The molecules of GlpG are colour coded according to the function of each structural element. TM helices 4 and 6 harbouring the active-site residues are shown in light blue; TM helices 2 and 5 that form the interface for substrate binding are in yellow; L1 is in pink; the extended loop, L3 upstream of the active-site serine that probably binds substrate, is in cyan; and TM helices 1 and 3 are in grey. L5 that covers the active site is in orange. (b) Two views of a surface representation of GlpG in a lipid environment. An almost complete bilayer around a GlpG molecule is depicted. The protein molecule is colour coded according to the biochemical properties of the amino acids: positive and negatively charged residues are shown in blue and red, polar residues are in light blue; and the rest in grey. The carbon atoms of lipids from the same molecule are shown as green spheres, and those from symmetry-related molecules are shown as yellow spheres. Water molecules are shown as blue spheres. The solid grey bars denote the position of the phosphodiester groups of the lipid molecules and mark the hydrophobic boundary.

The crystals of GlpG S201T obtained by mixing with lipids are type I membrane protein crystals, as described by Michel,^29^ which mimic a lipid bilayer. The protein molecules are arranged in layers with neighbouring molecules having an up and down orientation (Supplementary Fig. S3). Lipid molecules, TM1 and TM5 mediate many of the lateral contacts between protein molecules in the crystal (Fig. 3a). Adjacent membranous layers are held together by interaction between L1 and L4. In particular, Arg227 at the L4/TM5 boundary interacts with two acidic residues, Glu118 and Asp128, in L1 of two symmetry-related neighbours to hold the layers together (Fig. 3a). A total of 14 fully or partially ordered lipid molecules have been modelled in the current structure, and with contributions from symmetry-related lipid molecules, they form an annulus around the protein (Fig. 3b). Since the crystallisation method used involves a mixture of lipid and detergent, how can one be sure that the densities are indeed lipid molecules? The presence of connected density for two acyl chains in three of the modelled lipids unambiguously distinguishes them from detergent (Supplementary Fig. S4). The rest of the modelled lipid molecules do not have clear density for glycerol backbone or the head group and are presently modelled as single acyl chains of DMPC (see Supplementary Fig. S4 and Table 2). Since the detergent nonyl glucoside used to purify GlpG is still present in the crystallisation drop, the hydrocarbon chain of this detergent could also be modelled equally well to the densities corresponding to PC 504, 505, 508, 511, 513, and 514.

The average temperature factor for lipid molecules is double that of the protein molecule but considerably lower than those observed in bacteriorhodopsin^9^ or V-type ATPase.^12^ Most lipid molecules are clustered around TM1, L1, and TM3 in both leaflets. There is an asymmetry observed in the rest of the protein, with well-ordered lipids being found only on the cytoplasmic side, thus making the bilayer imperfect. The hydrophobic belt of GlpG inferred from the positions of aromatic and charged residues is ∼ 23 Å wide (Supplementary Fig. S5). Due to disordered head groups and the glycerol backbone in many of the lipid molecules, an exact thickness of the bilayer cannot be determined. However, by considering symmetry-related lipid molecules, an average bilayer thickness of ∼ 25 Å can be measured, which is close to the observed distance in the lamellar phase of DMPC bilayers.^30,31^ In the present structure, the lipid head groups and the C2 atoms of the glycerol backbones are at different depths around the protein, causing the bilayer thickness to vary. This could be because of packing constraints in the present crystal form (protein molecules arranged in an up and down orientation and lipid molecules mediating much of the lateral interaction in the same layer) or the presence of detergents disrupting the bilayer.

### Crystallisation of GlpG by detergent dialysis

**Fig. 4:**
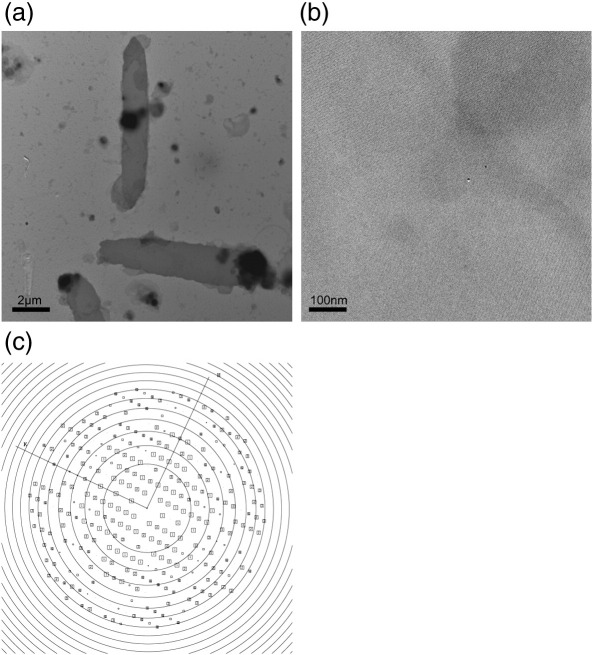
Two-dimensional crystals of GlpG. (a) Crystalline membranes of N-terminally truncated GlpG WT negatively stained with 1.5% uranyl acetate. (b) High-magnification image of a crystalline membrane showing the crystal lattice. (c) Calculated Fourier transform of a single image of GlpG with a rectangular unit cell of 37.6/56.4 Å. Each square on the reciprocal lattice describes a Fourier component, with the size and number of the square reflecting its signal-to-noise ratio.^34^ The largest boxes and the smallest numbers describe the most significant reflections. Concentric rings indicate the zero crossings of the contrast transfer function. Well-preserved and flat crystals show spots to ∼ 4 Å.

**Fig. 5:**
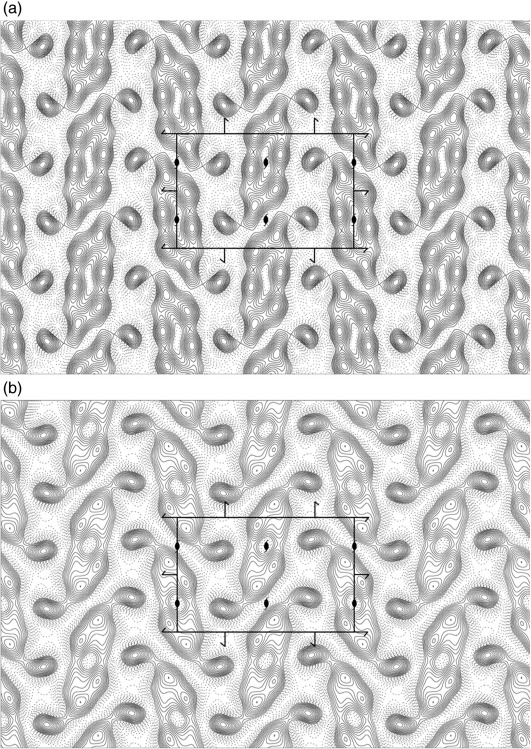
A comparison of maps obtained by electron and X-ray crystallography. (a) A projection map was calculated by merging five images of 2D crystals to 8 Å in *p*22_1_2_1_ symmetry by including spots to an IQmax of 4. Note that space groups *p*22_1_2_1_ and *P*2_1_2_1_2_1_ are indistinguishable in projection. The phases were averaged and rounded to 0° or 180°. Continuous lines indicate density above the mean, while negative contours are shown as dotted lines. An isotropic temperature factor (*B* = − 200) was applied to compensate (sharpen) for the resolution-dependent degradation of image amplitudes. The map was scaled to a maximum peak density of 250 and contoured in steps of 21. (b) A projection map of GlpG calculated from the PDB coordinates of GlpG S201T in the orthorhombic crystal form truncated to 8 Å. No temperature factor was applied, but the map was scaled and contoured as described for 2D crystals. For a direct comparison of the projection map with the packing in 3D crystals, please refer to Supplementary Fig. 3a. A unit cell is displayed with the *a*-axis vertical and the *b*-axis horizontal in both figures. It should be noted that the symmetry elements depicted in Fig. 5a are for the 3D space group *P*2_1_2_1_2_1_ and not for the 2D space group *p*22_1_2_1_ used to merge the images. It was obvious that these 2D crystals were thin 3D crystals rather than a single layer, and the fact that they have a similar packing to the 3D crystal obtained by vapour diffusion prompted me to use the same depiction of symmetry elements. The reason for the formation of stacked 2D arrays can be appreciated from the polar interaction observed between two layers mediated by charged residues between L1 and L4 (Fig. 3a).

Crystals of membrane proteins can also be obtained by reconstitution of detergent-solubilised proteins back into the lipid bilayer by gradual removal of detergents. This can result in 2D crystals.^32,33^ When GlpG was reconstituted into native *E. coli* polar lipids and detergent was removed by dialysis, crystalline membranes were obtained (Fig. 4a and b). These membranous crystals were well ordered and showed diffraction to ∼ 4 Å (Fig. 4c). However, they tended to form multilayers (i.e., they formed stacks of 2D ordered arrays). Analysis of these crystals shows that they belonged to the same orthorhombic crystal (*P*2_1_2_1_2_1_) form and had an almost identical unit cell to crystals obtained by the bicelle method. The relatively thick specimen results in a rapidly changing molecular transform. Together with the fact that secondary structural details in projection can be easily observed at intermediate resolution,^35^ this made it sensible to calculate a projection map of these 2D crystals at 8 Å (Fig. 5a). The projection map was then compared to an equivalent map computed from the X-ray crystallographic co-ordinates (Fig. 5b). The two maps are very similar, providing a strong argument that the protein molecules in the three-dimensional (3D) crystals obtained by the bicelle method are surrounded by lipids.

### Lipid–protein interaction

**Fig. 6:**
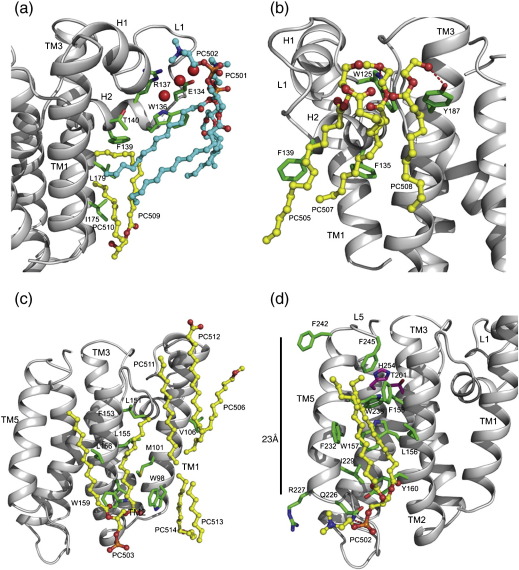
Lipid–protein interactions in GlpG. (a) Lipids PC509 and PC510 (yellow sticks) occupy the hydrophobic cavity created by the protrusion of L1. A number of contacts between the acyl chain and the hydrophobic side chains of TM1, TM3, and L1 (shown in stick representation and coloured green) are observed. Two-symmetry related lipids (cyan sticks) are found covering the H1 and H2 of L1. The present structure and the lipids around L1 explain the differential accessibility of the residues to cysteine-modifying reagents in a detergent or lipid environment observed by Wang et al.^36^ (b) Three lipid molecules occupy the V-shaped gap created by L1 between TM1 and TM3. The polar interactions between the oxygen atoms of the glycerol backbone of lipids PC507 and PC508 (red dotted line) are with the indole nitrogen of Trp125 and hydroxyl of Tyr187, respectively. (c) The lipid molecules along TM1 are spaced apart to give a hydrophobic thickness of ∼ 32 Å. The acyl chains 513 and 514 extend beyond the disordered N-terminus in the present structure. PC503 occupies the groove formed by TM1 and TM2. PC511 and PC512 are probably part of same lipid molecule and likely to bend over TM1 and L1. The acyl chain of PC506 interacts with hydrophobic residues of TM1. (d) Lipid PC502 occupies the interface between TM2 and TM5. A hydrogen bond is formed between the oxygen atom of the phosphodiester group and the side-chain amide of Gln226, and numerous hydrophobic interactions between the acyl chain and the side chains of residues along TM2 and TM5 are observed. The acyl chain of PC502 extends beyond the midpoint of TM helices 2 and 5. The terminal methyl group is only 4.4 Å from the side chain of Phe245 in L5, indicating that the side chains of this loop are clearly within the hydrophobic region of the bilayer. The solid black bar denotes the length of TM5 (∼ 23 Å) measured from Arg227 to Phe242.

Most of the lipid molecules modelled in the present structure are annular lipids (i.e., their acyl chains and the head group are in direct contact with the protein). The acyl chains fit nicely into grooves and crevices in the protein molecule. In the structure of GlpG, L1 exists as a separate entity protruding away from the TM helices and comprising two short α-helices, H1 and H2, which lie parallel to the membrane. Such an arrangement creates a hydrophobic cavity underneath the loop and a gap between TM1 and TM3. Two partially ordered lipid molecules occupy this cavity (Fig. 6a). In particular, the acyl chain of PC509 molecule bends into this cavity to interact with the hydrophobic side chains of amino acids from L1 and TM3.

The conserved ‘WR’ motif in L1 is characteristic of rhomboid proteases and is crucial for protease activity.^24,36^ This motif is part of H2 in L1, where the Trp136 side chain faces the membrane interior and the Arg137 side chain points into the protein to form numerous interactions with neighbouring residues including Glu134. Two symmetry-related lipid molecules plug the space around the WR motif (Fig. 6a). Water-mediated hydrogen bonds could potentially form between the side chains of Glu134 and Arg137 and the glycerol backbone of the symmetry-related lipid molecule (PC502), but are not observed in the present structure. A lipid molecule with a smaller head group than choline might be able to penetrate and interact with Arg137 and Glu134. Indeed, molecular dynamics simulations of GlpG in phosphatidyl ethanolamine membranes suggest that such interaction may be possible.^25^ The acyl chain of this symmetry-related lipid ends in the cavity created by L1. Three additional lipid molecules occupy a V-shaped gap between TM1 and TM3 (Fig. 6b). This unique arrangement shows how L1 can interact with lipid molecules, and suggests that it may be designed as a supporting element for substrate binding and catalysis.

Lipid molecules around TM1 are spaced farthest apart. There are three acyl chains in the periplasmic leaflet near TM1 and two acyl chains in the cytoplasmic leaflet. It is possible that some of these acyl chains (Table 2) are part of the same lipid molecules, but due to weak density, they have been modelled as separate chains. The positions of PC511 and PC512 indicate that they are likely to interact with H1 of L1, in particular with the side chain of Gln112 (Fig. 6c). PC513 and PC514 in the cytoplasmic leaflet probably extend beyond the disordered N-terminus, which includes residues Arg92 and Glu91. It is likely that these residues interact with the glycerol backbone and probably the phosphodiester oxygen atoms of the lipid. In the present model, the distance measured across the membrane between these lipids is ∼ 32 Å, which is larger than the average width of the hydrophobic belt around GlpG. The groove formed between TM1 and TM2 is occupied by the acyl chain of PC503 and interacts with the hydrophobic side chains of amino acids from both these helices.

A well-ordered lipid molecule, PC502, fits nicely into the groove created by helices TM2 and TM5. The distal tip of the acyl chain is only 4.4 Å from the side chain of Phe245 in L5, confirming the proposal that the side chains of residues from L5 are in the hydrophobic region of the bilayer^36^ (Fig. 6d). The length of TM5 is ∼ 23 Å, with the fully extended acyl chain PC502 traversing more than half of this length. Since there are no lipid molecules on the periplasmic leaflet around this region, it is not easy to determine the exact thickness of the bilayer. It is likely that in the region around TM5, lipids from the two leaflets probably interdigitate to match the local hydrophobic thickness of the protein. The structure of GlpG in a lipid environment thus provides insights into the adaptation of lipids to the surface features of the protein.

### Substitution of lipids by detergent molecules

**Fig. 7:**
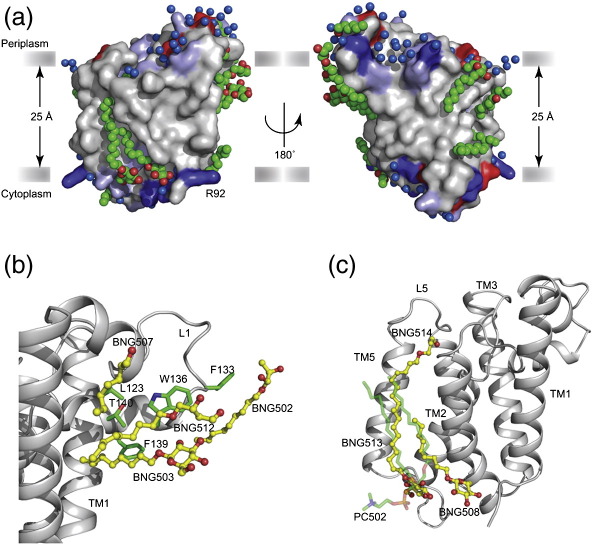
Substitution of lipids by detergent. (a) Surface representations showing two views of GlpG S201T in the trigonal crystal form with a detergent environment. The protein molecule is coloured as in Fig. 3b. Some detergent molecules (carbon atoms as green spheres) are observed in analogous positions to lipid molecules. Water molecules (blue spheres) and the solid grey bars mark the membrane boundary. The arginine (R92) residue at the N-terminus of the protein is marked to show the region that is disordered in the structure determined in a lipid environment. (b and c) Interactions of selected detergent molecules with the protein. Detergent molecules are shown as yellow stick representations; the side chains of amino acids interacting with detergent are shown in green; and the backbone of the protein molecule is in grey. Panel (b) shows four detergent molecules found interacting with residues in L1. The hydrophobic cavity created by L1 is filled with two of these molecules but in contrast to the position of the lipid (Fig. 4a); the head groups of these detergents are in direct contact to some of the residues. Panel (c) shows the detergent molecules BNG508 and BNG514 at the TM2/TM5 interface stretching from the cytoplasmic end of TM2 to L5 at the periplasmic end. Together, these two detergents occupy the same position as the sn1 acyl chain of the lipid molecule PC502 (shown as transparent green sticks), while BNG513 substitutes for the sn2 acyl chain.

The structures of GlpG S201T in detergent and lipid environments provide a unique possibility to study how artificial detergents substitute for lipids. Of the 18 fully or partially ordered detergent molecules in the trigonal crystal form of GlpG S201T, many take similar positions to lipids, binding in grooves and crevices in the protein and demonstrating how the native structure of GlpG is preserved in a detergent environment (Fig. 7a and Table 3). The average temperature factors of detergent molecules are slightly higher than those of lipids, indicating a higher disorder or mobility. Six detergent molecules surround L1 and two occupy the space beneath it, thus preserving the structure of L1 (Fig. 7b). There are three well-ordered detergent molecules at either end of the TM2/TM5 interface. Detergent BNG508 in the cytoplasmic leaflet traverses from the bottom of TM2, with its hydrophobic chain running along TM2 to meet the end of BNG514, which is positioned in the periplasmic leaflet (Fig. 7c). Together, these two detergents span ∼ 17.3 Å (measured from C1–C1 atoms) and occupy a similar position to the sn1 acyl chain of the lipid molecule PC502. Another detergent, BNG513, occupies the position of the sn2 acyl chain of PC502. Thus, it is reassuring to see that detergent provides an excellent substitute for lipids by preserving the global architecture of GlpG. However, lipids are likely to be essential for modulating the catalytic activity.^37^

### Detergent mimics substrate binding

**Fig. 8:**
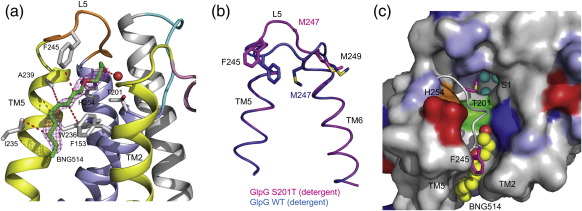
Detergent mimics substrate binding. (a) BNG514 is well ordered in the present structure as observed in the *F*_o_ − *F*_c_ difference map (magenta mesh) calculated before modelling of the detergent, contoured at 3σ. The protrusion of BNG514 causes minor displacement of L5 residues, in particular Phe245 and Met247. There are numerous hydrophobic as well polar contacts between BNG514 and residues from TM2, TM5, and L5. If modelled, the head group of BNG514 is only ∼ 4 Å from the threonine. (b) An overlay of GlpG WT and S201T structures showing partial displacement of L5. The side chain of Met247 is disordered in the GlpGS201T structure. The different conformations of L5 are functionally important because, in the WT structure, the two side chains of methionine point into the active site; for peptide hydrolysis, this loop needs to move away. With one of the methionine residues still occluding the GlpG S201T active site, it is possible that it reflects an intermediate conformation of L5. (c) Top view of the GlpG S201T molecule in surface representation (coloured as in Fig. 3b) to show the bending of detergent (yellow spheres) towards the active site. The active-site residues are coloured green (Thr201) and orange (His254). L5 is shown in cartoon representation, with side chains of Phe245 and Met249 highlighted in magenta. Three water molecules (cyan spheres) are found in a cavity, which was predicted as the substrate binding S1 site.^26^ The side chain of Met249 points into the cavity.

BNG514 observed in the trigonal crystal form occupies the space between TM2/TM5 and protrudes into the active site (Fig. 8a). Such an insertion of detergent results in minimal structural change involving only a partial displacement of L5 in particular residues (residues 245–247; Fig. 8b and Supplementary Fig. S6a). The side chain of Met249 still points into the active site as in the WT structure (Fig. 8b). The head group of this detergent is disordered but can be accommodated in the space very close to the active site. When modelled, the oxygen atom of the head group is ∼ 4 Å from Thr201. What is the significance of this detergent? Hydrophobic substrate TM proteins are believed to diffuse laterally in the bilayer and to partition or interact with the enzyme through the gap between TM2/TM5.^23,38^ Reinforcing this notion, a hydrophobic lipid or detergent molecule is observed in both the structures described here (Figs. 6d and 8c). The presence of glycine or proline residues in the substrate at or near the cleavage site has been found to be essential for efficient cleavage of rhomboid substrates.^39–41^ Glycine and proline are residues that are known to introduce kinks in TM helices, and it is probable that the TM helix of the substrate must bend towards the enzyme thereby juxtaposing the cleavage site of the substrate with the active site of enzyme. This detergent molecule may be mimicking a rhomboid substrate bending towards the active site (Fig. 8d).

A partially ordered detergent molecule is observed in the same position as BNG514 in the GlpG WT structure, where it remains fully extended and does not bend into the active site (Supplementary Fig. S6b). Why and how this detergent protrudes into the active site only in the S201T structure is not clear, since the structures of WT and S201T in a detergent environment are very similar (Fig. 1b). Due to their inherent flexibility, it is possible that two functionally relevant conformations of L5 have been captured in the structures of the WT and S201T mutant (Fig. 8b).

## Discussion

With the structure of GlpG in a lipid environment, two important conclusions can be made. First, the largely identical structures of GlpG determined in a lipid or detergent environment validates the use of detergents as a good mimic for substituting lipids, at least in the case of GlpG. Second, the bilayer thickness around GlpG varies across the protein, and lipid molecules adapt to match the local environment of the protein. An idealised view of a membrane protein in a bilayer pictures the acyl chains of the lipid molecules as straight with the head groups at constant depth. The structures of bacteriorhodopsin and Aqp0 in a membranous environment support this view. However, these proteins form trimeric or tetrameric assemblies that then crystallise into ordered 2D arrays, thereby constraining the packing of lipids around the protein.^8–11^ Similar constraints due to crystal packing observed in the orthorhombic crystal form of GlpG restrict the mobility of lipid molecules, and the lipid molecules adapt to the surface features of GlpG. From the present model, it seems that the depth of head groups is not uniform and bilayer thickness varies. A similar scenario was observed in the structure of a voltage-gated potassium channel determined in a lipid-like environment.^13^ One possibility arising from that structure was that a mixed micelle of detergent and lipid might not form ideal membranes. However, as shown from the map obtained from 2D crystals (obtained by detergent removal), it is likely that the present structure of GlpG closely resembles that in native membranes. There may still be differences between how the different lipids used in 2D and 3D crystals interact with rhomboid. Analogous comparisons of Aqp0 show that the structure is largely identical in different lipid environments, with differences observed in the way the head groups interact or the longer acyl chains interdigitate on the surface of the protein.^11^

On the basis of the high-resolution structure of GlpG^36^ and the molecular dynamics simulation in fluid bilayers,^25^ it has been proposed that bilayer thickness in the immediate vicinity of the protein is thinner. The present structure with its static view of lipids around GlpG does indicate the possibility of membrane thinning, in particular around TM5. Two detergent molecules are found on either side of the protein at the TM2/TM5 interface (Fig. 7c); with an acyl chain length of 9, they cover almost the entire TM5. This also indicates that in *E. coli* inner membranes with an average acyl chain length of 16–18, lipid molecules will need to adapt to match the local hydrophobic thickness. Approximately 55% of the lipids in *E. coli* inner membrane are unsaturated,^42^ and the presence of unsaturated lipids is known to alter the bilayer thickness, typically making it 2 to 3 Å thinner than with a saturated lipid of similar length.^30,43,44^ In the recent structure of Aqp0 in *E. coli* polar lipids, the average C2–C2 distance of glycerol backbone was measured as ∼ 27 Å^11^. Thus, it is possible that a small compression of bilayer is required to match the local hydrophobic thickness in the vicinity of TM2/TM5.

**Fig. 9:**
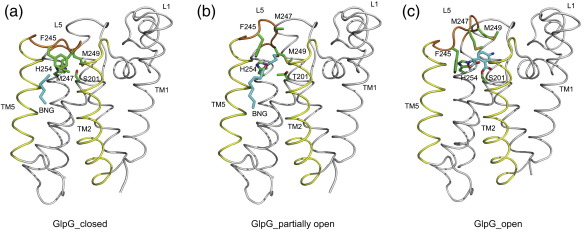
Three experimentally determined structures that provide a sequential picture, and therefore a model, for substrate binding and catalysis in rhomboid protease. The structural changes required for substrate binding and catalysis can be followed by changes in loop 5 (backbone coloured in orange) and the side chains (shown as green sticks) within it. The active-site residues Ser/Thr201 and His254 are shown as green sticks. Substrate is likely to partition into the enzyme through TM helices 2 and 5 (shown in yellow). The rest of the molecule is shown in grey. (a) The closed state of GlpG (PDB code 3B45 or 2XOV), as defined by the inward pointing of side chains of Met247 and Met249 in loop 5, prevents binding at the active site. A partially ordered detergent molecule (cyan sticks and labelled BNG) is found at the TM2/TM5 interface. (b) A partially open state of GlpG (PDB code 2XTU) is represented by the protrusion of a detergent molecule (cyan sticks) with a displacement of residues 245–247 in loop 5. The side chain of Met249 still points into the active site, thus indicating that this needs to make way for binding at the active site. (c) The open state with an inhibitor (cyan) bound covalently to the active residues (PDB code 2XOW) shows that both Met247 and Met249 are lifted away from the active site. This is accompanied by a small displacement of TM5.

The structures of the native and acyl enzyme of GlpG have provided insights into the global architecture of rhomboid protease and suggested a probable mechanism for substrate recruitment and proteolysis^23,26,38^. With the new structures described in this study, it is now possible to extend our understanding about how a substrate might partition into the active site of rhomboid protease. As described previously,^19,26^ the closed state of GlpG is characterised by the side chains of Met247 and Met249 in L5, pointing into and blocking access to the active site (Fig. 9a). For a substrate to bind and be cleaved, both these bulky methionine side chains need to move away from the active site. The first step in such a process might involve substrate interaction through the TM2/TM5 interface and movement of L5. The structures of GlpG S201T and detergent BNG514 provide a clue about how the substrate might partition into the active site. A small displacement of L5 lifts the side chains of Phe245 and Met247, but Met249 in the present structure still plugs the cavity near the active site (Fig. 9b). This probably represents an intermediate state of the enzyme during substrate binding. For a substrate to be able to bind at the active site, the side chain of Met249 also needs to move out, and the structure of GlpG in complex with a covalently bound isocoumarin inhibitor^26^ shows this is indeed the case, and therefore could represent an open state (Fig. 9c). Binding of inhibitor is accompanied by only a small displacement of TM helix 5. These structures thus show that a large structural change is probably not essential for substrate binding and proteolysis by GlpG. From a structural point of view, the challenge to crystallise the complex of a full-length TM protein substrate with a rhomboid remains. Knowledge of this structure is essential to validate how the substrate binds and the extent of structural change required for hydrolysis. With tools to crystallise GlpG in detergent as well as with lipids, the goal of obtaining a structure of GlpG with a peptide should be achievable.

## Materials and Methods

### Protein expression and purification

The active-site mutation of S201T was performed with the QuickChange method (Stratagene). Full-length GlpG in pET25b vector with a C-terminal His-tag was expressed in *E. coli* BL21 C41 (DE3) cells in 2YT medium. Membrane preparation, purification by metal affinity chromatography, removal of the N-terminal domain of GlpG, ion-exchange chromatography, and gel filtration chromatography were carried out as described previously.^26^

### 3D crystallisation

Crystals of completely delipidated, truncated GlpG S201T were obtained by mixing a solution of 2.5–3.0 M ammonium chloride with protein (∼ 8 mg/ml in n-nonyl-β-D-glucoside) at a ratio of 1:1 in hanging drops at 25 °C. For bicelle preparation, DMPC powder (Avanti polar lipids) and CHAPSO (Glycon) at a ratio of 2.6:1 were dissolved in water to give a final combined concentration of 20% DMPC/CHAPSO. Based on previous experience with 2D crystallisation, different protein-to-bicelle ratios were tried, ranging from a ratio of 10:1 to 2.5:1 (protein/bicelle, w/w). Prior to crystallisation, protein at a concentration of 10 mg/ml in nonyl glucoside was mixed with bicelles to give final concentrations of protein (∼ 8–9.5 mg/ml) and bicelles (1–4%) and incubated on ice for 1 h. Crystals of GlpG S201T in bicelles were obtained under polyethylene glycol-based or salt-based conditions. However, the best diffracting crystals were obtained with a final protein concentration of 9 mg/ml and 2% bicelles mixed with 1.5 M NaCl, 0.1 M Bis-Tris (pH 7) at a ratio of 1:1 in hanging drops at 25 °C. The crystals started appearing after a week and grew to a maximum size of 0.3 × 0.08 × 0.05 mm over a period of 20–30 days. All crystals were cryo-protected by adding 25% glycerol to the mother liquor and rapidly frozen in liquid nitrogen.

### X-ray crystallography

Data sets were collected on the I03 beam line at the Diamond Light Source (Harwell). Diffraction data were indexed and integrated with imosflm and reduced with SCALA.^45,46^ The structure of WT GlpG in the trigonal crystal form [Protein Data Bank (PDB) code 2XOV], with residues 245–249 corresponding to L5 omitted, was used as an initial input model for Phaser.^47^ After modelling the residues in L5, rigid-body and restrained refinement was carried out with PHENIX,^48^ followed by manual model building in COOT.^49^ For lipid molecules with the strongest density, the complete acyl chains were directly built. Using a 3*F*_o_ − 2*F*_c_ map at 1σ as a guide, lipid molecules with weak densities were built towards the end of the refinement as short acyl chains and were extended as phases improved. The final model of GlpG S201T in the orthorhombic crystal form includes residues 92–245 and 248–272 (the densities for 246 and 247 were weak and not modelled), 14 lipid molecules, and 79 water molecules. The model for GlpG S201T in the trigonal crystal form was similarly refined. The final model contains residues 91–271, 18 detergent molecules, and 87 water molecules.

### 2D crystallisation, electron microscopy, and image processing

Truncated GlpG WT in 0.2% n-Decyl-β-D-maltopyranoside (DM) at a final concentration of 1 mg/ml and *E. coli* polar lipids (5 mg/ml) (Avanti Polar Lipids) solubilised in 1% DM were mixed at a lipid-to-protein ratio of 0.45–0.55 (w/w) and incubated at room temperature for 1 h before being transferred to dialysis bags or Slide-A-lyzer MINI dialysis units (PIERCE) with a  10-kDa cutoff. Dialysis for detergent removal was carried out at 37 °C in 25 mM Bis-Tris (pH 7), 0.05 M NaCl, 5% glycerol, 5% 2-4-methylpentanediol, 3 mM NaN_3_, and 1 mM DTT for 7 days. The harvested crystals were stable for several months at 4 °C.

Samples after dialysis were analysed for crystallinity by negative staining with 1% uranyl acetate and screening with a Tecnai 12 electron microscope. For electron cryomicroscopy, continuous carbon on a copper/rhodium grid that had been glow discharged in air for 30 s was used to analyse batches of well-ordered crystals that had been identified by negative staining.  The sample (2–4 μl) was applied to a grid held in an FEI Vitrobot under a controlled atmosphere of 18 °C and 80% humidity. Grids were blotted for 7 s and rapidly frozen in liquid ethane. Images were recorded in a Polara G2 electron microscope at an accelerating voltage of 300 kV and a magnification of 59,000×, with the specimen at liquid nitrogen temperature. Images recorded on a Kodak SO-163 electron emulsion film were developed for 12 min in a full-strength Kodak D19 developer. The quality of the negatives was evaluated by optical diffraction for the absence of astigmatism and tilt, and those exhibiting strong reflections to 6–8 Å were selected for further processing. The whole negative was digitised with a pixel size of 6 μm on a KZA scanner^50^ and manually inspected using the interactive FFT option in the Ximdisp program.^51^ The best-ordered area of 4000 × 4000 pixels was selected using the program LABEL and further processed using the MRC image processing program to correct for lattice distortions and contrast transfer function.^34,52^ Typically, two cycles of unbending were carried out for each image. The program ALLSPACE was used to determine the symmetry and phase residuals.^53^ The five best images were merged at 8 Å to generate a data set from which a projection map was calculated. To calculate the projection of the X-ray structure, structure factors were calculated from the coordinates and a projection map was calculated with the same parameters as for the 2D crystals, except that no *B*-factor sharpening was applied.

### Rhomboid activity assay

The TM domain of the artificial substrate LacY TM2 was expressed as a fusion protein with an N-terminal maltose binding protein domain, a C-terminal thioredoxin domain, and a His-tag (construct pKS35^41^) in *E. coli* C41 (DE3). Expression, membrane preparation, solubilisation, and purification with nickel affinity column were carried out as described previously.^26^ A typical rhomboid assay was carried out in 25 mM Tris buffer (pH 8), 0.15 M NaCl, and 0.2% DM in a final volume of 10 μl and incubated at 37 °C for 2 h. Cleavage products were separated on a 10% Bis-Tris gel (Invitrogen) developed with Mes buffer, and bands were stained with Coomassie brilliant blue.

### Protein Data Bank accession number

The coordinates and structure factors of the GlpG S201T structures in lipid and detergent environments have been deposited at the PDB with accession numbers 2XTV and 2XTU.

The following are the supplementary materials related to this article.

Supplementary materials

## Figures and Tables

**Table 1:** Data collection and refinement statistics

	GlpG S201T (lipid environment)	GlpG S201T (detergent environment)
*Data collection*		
Space group	*P*2_1_2_1_2_1_	*R*32
Cell dimensions		
a, b, c (Å)	38.6, 58.8, 91.1	110.2, 110.2, 127.6
Resolution (Å)	45.5–1.70 (1.79–1.70)^a^	42.5–1.85 (1.95–1.85)^a^
*R*_merge_	0.049 (0.306)	0.089 (0.573)
I/σI	13.3 (3.2)	12.2 (2.8)
Completeness (%)	95.8 (86.9)	99.9 (99.9)
Redundancy	3.3 (3.1)	5.3 (5.4)

*Refinement*		
Resolution (Å)	28.0–1.7 (1.77–1.7)^a^	38.2–1.85 (1.92–1.85)^a^
No. of reflections	22,432	25,579
*R*_work_/*R*_free_^b^	0.186/0.215 (0.228/0.26)^a^	0.176/0.198 (0.22/0.25)^a^
No. of atoms		
Total	1788	1757
Protein	1413	1459
Lipid	296	—
Detergent	—	211
Water	79	87
*B*-factors (Å^2^)		
Total	24.1	28
Protein	19.9	24.3
Lipid	41.9	—
Detergent	—	50.0
Water	31.7	36.1
R.m.s. deviations		
Bond lengths (Å)	0.006	0.006
Bond angles (°)	1.02	0.997
Maximum likelihood coordinate error (Å)	0.19	0.18

a Values in parentheses are for the highest-resolution shell.

b *R*_free_ was calculated using a randomly selected subset of reflections (5%); the remaining (95%) reflections were used for the calculation of *R*_work_.

**Table 2:** Summary of modelled lipids in the *P*2_1_2_1_2_1_, crystal form

Lipid	Position^a^	Polar interaction^b^	Density for			*B*-factor (Å^2^)
			Head group^c^	Acyl chain^c^		
				C11–C24	C31–C44	
501	Cytoplasmic, TM6	R217	+++	+++ (8)	+++ (8)	33
502	Cytoplasmic, TM2/5	Q226	++	+++ (14)	+++ (14)	40.3
503	Cytoplasmic, TM2/1	—	+	++ (14)	++ (14)	43.3
504	Periplasmic, TM6	—	−	−	++ (11)	45.9
505	Periplasmic, L1	—	−	++ (14)	+ (4)	45.6
506	Periplasmic, TM1/L1	—	−	−	++ (14)	39.9
507	Periplasmic, TM1/3	W125	−	++ (11)	−	41.1
508	Periplasmic, TM3	Y187	−	−	+ (11)	47.4
509^d^	Cytoplasmic, cavity underneath L1	—	−	+ (14)	−	44.6
510^d^	Cytoplasmic, cavity underneath L1	—	−	−	+ (9)	40.8
511^d^	Periplasmic, TM1	—	−	+ (14)	−	43.8
512^d^	Periplasmic, TM1	—	−	−	+ (14)	43.1
513^d^	Cytoplasmic, TM1	—	−	−	+ (8)	45.2
514^d^	Cytoplasmic, TM1	—	−	+ (14)	−	41.6

a Position defines whether the lipid is present at the periplasmic or cytoplasmic leaflets.

b Polar interactions within 3.5 Å between the oxygen atoms of the phosphodiester group or glycerol backbone of the lipid molecule and the charged residues or hydroxyl groups or side-chain amine or indole nitrogen of the protein molecule.

c Density for the head group (choline and phosphodiester) and the acyl chains. +++, very good density; ++, moderate density; +, weak density; −, no density. Numbers in parentheses denote the number of carbon atoms built for each acyl chain.

d These acyl chains (509 and 510, 511 and 512, 513 and 514) could be part of the same lipid molecule, but the present map does not allow for unambiguous modelling as a single lipid molecule.

**Table 3:** Summary of modelled detergents in the *R*32 crystal form

Detergent	Position^a^	Polar interaction^b^	Density for		*B*-factor (Å^2^)
			Head group^c^	Hydrocarbon chain^c^ C1–C9	
501	TM5/TM6	—	−	+ (9)	51.6
502	L1	—	−	+ (9)	44.4
503	Underneath L1	—	+	+ (9)	59.9
504	Underneath L1	—	−	+ (7)	43.2
505	TM5/TM6	—	−	+ (7)	43.1
506	TM5/TM6	—	−	+ (7)	39.6
507	L1	—	−	+ (7)	46.8
508	TM2/L4/TM5	Y160, A93, E91	+	+ (9)	45.3
509	L1, on sym axis	—	−	+ (6)	40.2
510	L1	T130	−	+ (6)	45.9
511	TM5/6	—	−	+ (6)	51
512	Underneath L1	—	−	+ (9)	54.1
513	TM2/TM5	Q226	+	+ (9)	63.4
514	TM2/TM5	—	−	+ (9)	44.8
515	L4	G215	−	+ (4)	55.9
516	TM1	—	−	+ (2)	37.5
517	TM6	N251	+	+ (9)	52.9
518	TM1	R92	−	+ (9)	45.1

a Position defines where the detergent molecule is observed.

b Polar interactions within 3.5 Å between the oxygen atoms of the head group of the detergent molecule and the protein molecule involving mainly side-chain hydroxyls, side-chain amides, or backbone carbonyl and amides.

c +/− denotes if the density for the head group (glucoside) or the hydrocarbon chain was present. Numbers in parentheses denote the number of carbon atoms built for each acyl chain.
