# Supplementary material for: Structure of Rhomboid Protease in a Lipid Environment
Source: J Mol Biol. 2011 Mar 25;407(2):232–47. doi: 10.1016/j.jmb.2011.01.029 (PMC3093617; doi:10.1016/j.jmb.2011.01.029)
Supplement: Supplementary file 1 — Supplementary materials [file mmc1.pdf]

Structure of rhomboid protease in a lipid environment

Supplementary material

Kutti R. Vinothkumar*

MRC Laboratory of Molecular Biology, Hills Road,
Cambridge CB2 0QH, UK.
vkumar@mrc-lmb.cam.ac.uk
Tel: 44-1223-402405
Fax: 44-1223-213556

Supplementary figure legend:

Supplementary figure 1: A comparison of temperature factors of GlpG S201T in a lipid and detergent environment

A) A cartoon representation of GlpG S201T determined in a lipid or detergent environment. The spectrum of colour denotes, blue as having the lowest B factor and orange/red as having the highest B factor.

B) A graphical display of the average B factor distribution of all protein residues. It is very clear that major difference in temperature factor is found in the loop regions in particular L4 and L5. In L4 residues Arg217 and Gln226 have substantially lower temperature factors of 17.9 $Å^2$ and 28.6 $Å^2$ respectively in the lipid environment as opposed to 41.5 $Å^2$ and 43.5 $Å^2$ in presence of detergents. This is possibly because of their restricted mobility brought by their interaction with the lipid head groups and the crystal contact between Arg227 with acidic residues from L1 of symmetry related molecules (Fig 3A).

Supplementary figure 2:

Stereo view of a 2Fo-Fc map (blue mesh) contoured at 1σ and a Fo-Fc map (magenta mesh) at 3σ after final refinement showing the unmodelled density at the active site.

Supplementary figure 3: Type I membrane protein crystals of GlpG.

Packing in the orthorhombic, membranous crystal form of GlpG obtained by the bicelle method. Views along the c and a axis are shown in panels A and B respectively. Only TM5 (yellow) and L1 (red) are coloured as a guide to show the orientation of the molecule. In panel B the side chains of Glu118, Asp128 (red sticks) and Arg227 (blue sticks) that mediate the interaction between two layers are highlighted. The up and down orientation of GlpG molecules and the shift along the a-axis in the same layer can be best seen in panels B and A respectively.

Supplementary figure 4:

The 2Fo-Fc map is shown in panels are contoured at 1σ for all lipid molecules described in the present model.

Supplementary figure 5: A simplistic representation of GlpG in a lipid environment. The backbone of GlpG is shown in grey, lipid molecules as yellow sticks, water molecules inside the protein as blue spheres and on the surface as red spheres. The side chains of residues that could mark the hydrophobic boundary are shown as green sticks and labelled. To describe the amphiphilic nature of L1, key residues including Arg137, Trp136, Phe135 and Phe139 are shown. The active site residues are shown as magenta sticks. A typical bilayer consists of distinct regions that can be divided into the hydrophobic core consisting of acyl chains, the interface layer with the phosphodiester group and probably glycerol backbone and the aqueous solution. In the present structure of GlpG, the phosphodiester group of many lipids is disordered but by using the charged amino acid residues as a guide, the black bar in the figure has been drawn at the height of the phosphodiester groups to mark the hydrophobic boundary. An independent estimation of the hydrophobic thickness of GlpG molecules based on the aromatic and charged residues also gives an average value of ~23 Å. The idea for this representation was inspired from the depiction by Wang et al 2007 and White et al 2002.

Supplementary figure 6:
A) Stereo view of a 2Fo-Fc map contoured at 1σ showing the density of L5, corresponding to residues 244-250 and the side chain of Met249 pointing into the active site.
B) Top (periplasmic) view of crystallographic trimer of GlpG S201T and WT with detergent molecules at TM2/TM5 interface highlighted as red sticks. The protein molecule is colour coded as in figure 3A of the main text. In the GlpG S201T structure, BNG514 bends into the active site with a small displacement of L5. A partially ordered detergent molecule is found in the same position in GlpG WT structure but it is not bent.

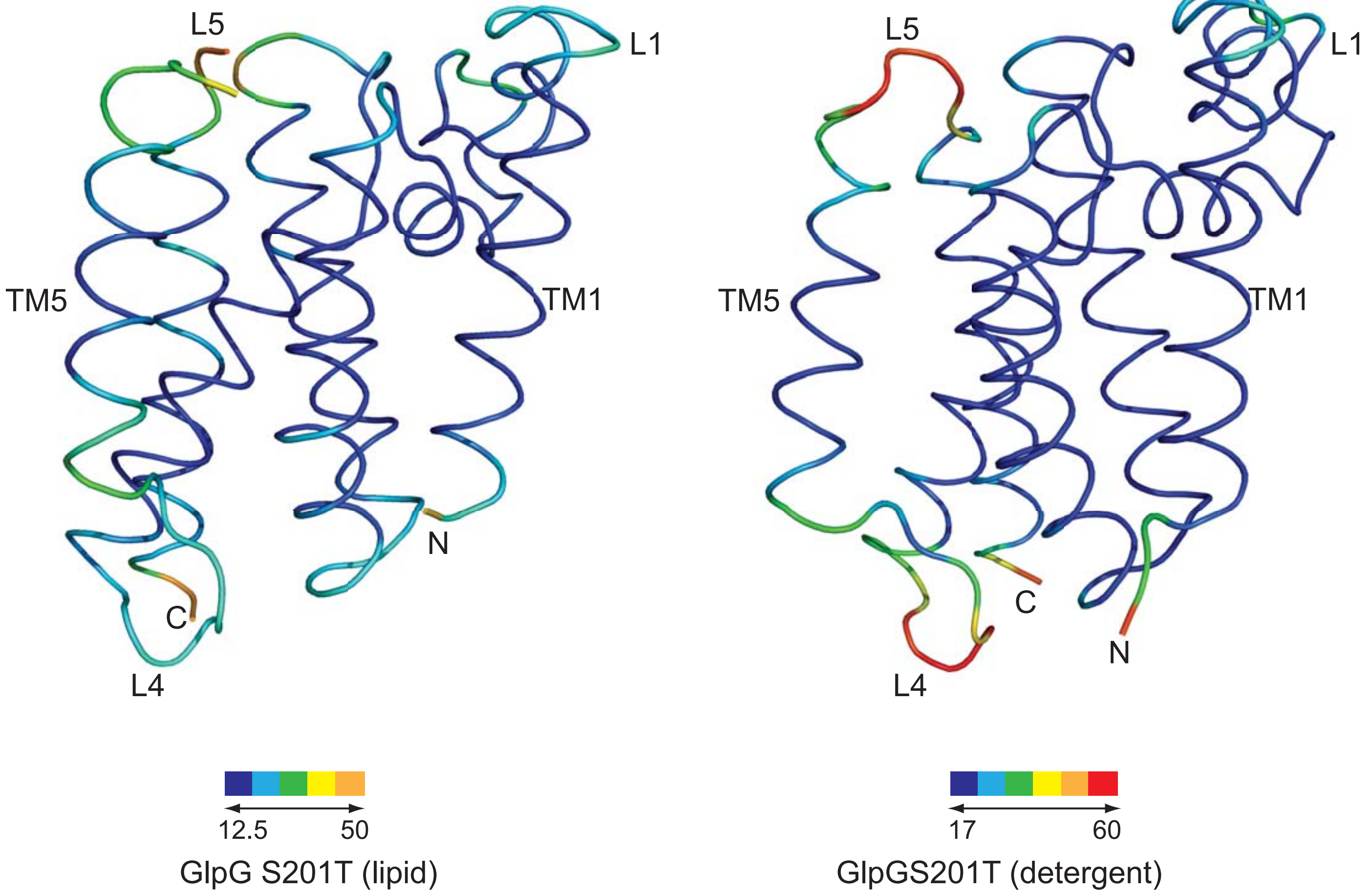

(A)

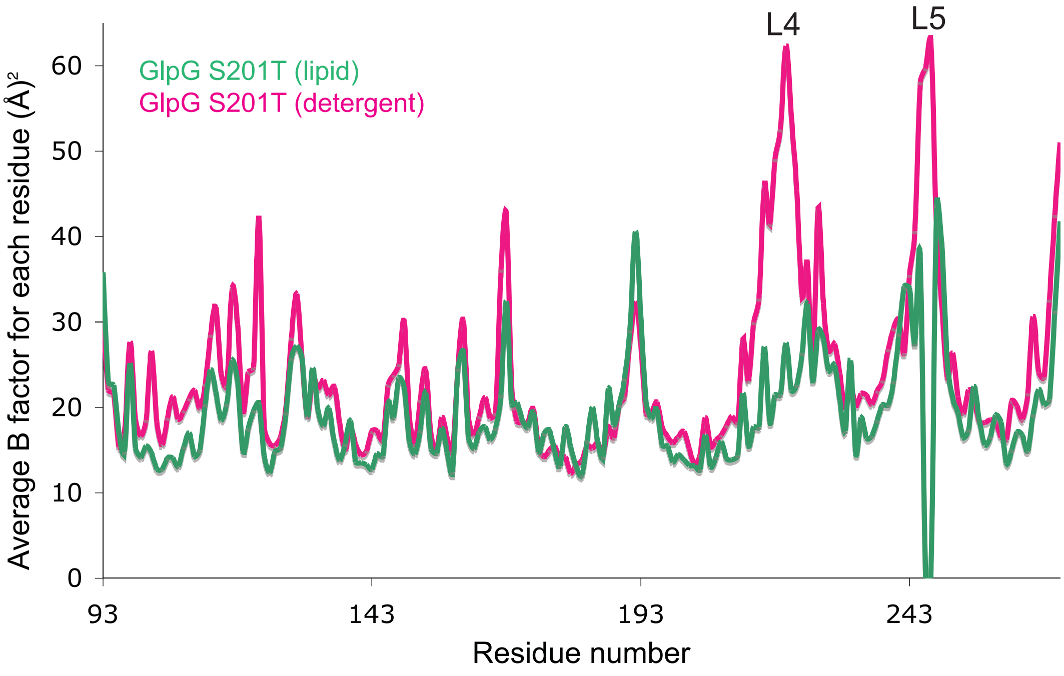

(B)

Supplementary Figure 1

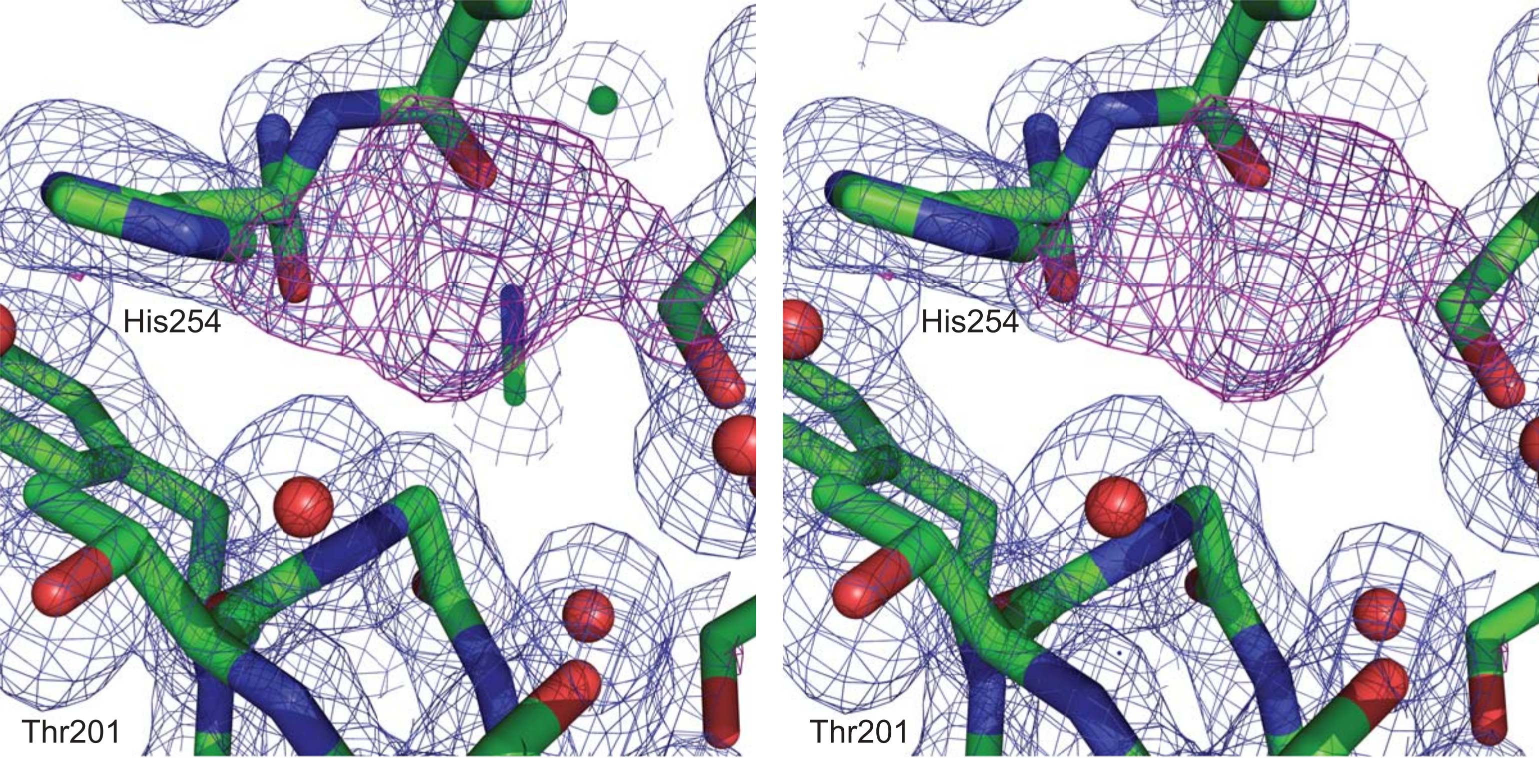

Supplementary figure 2

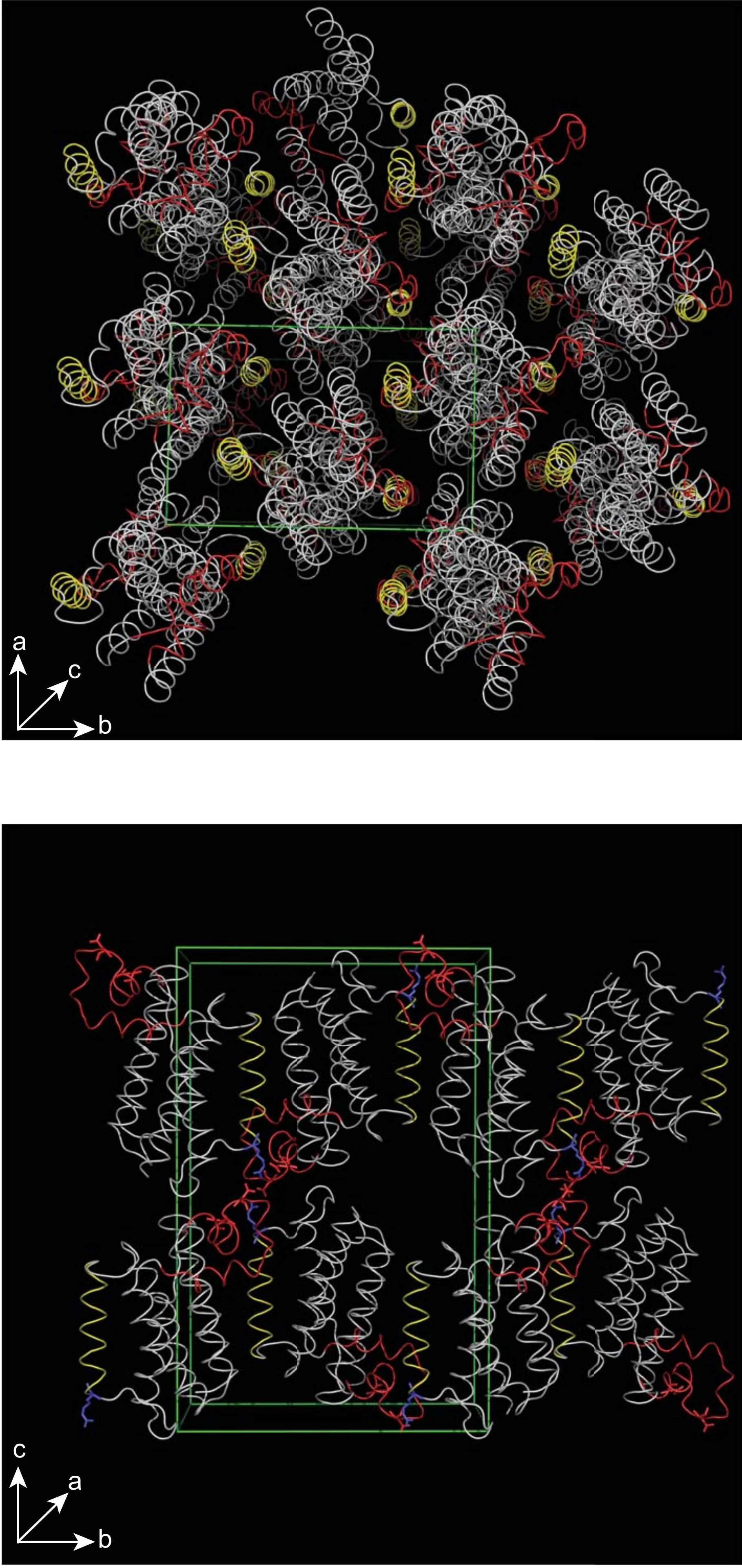

A)
B)

Supplementary figure 3

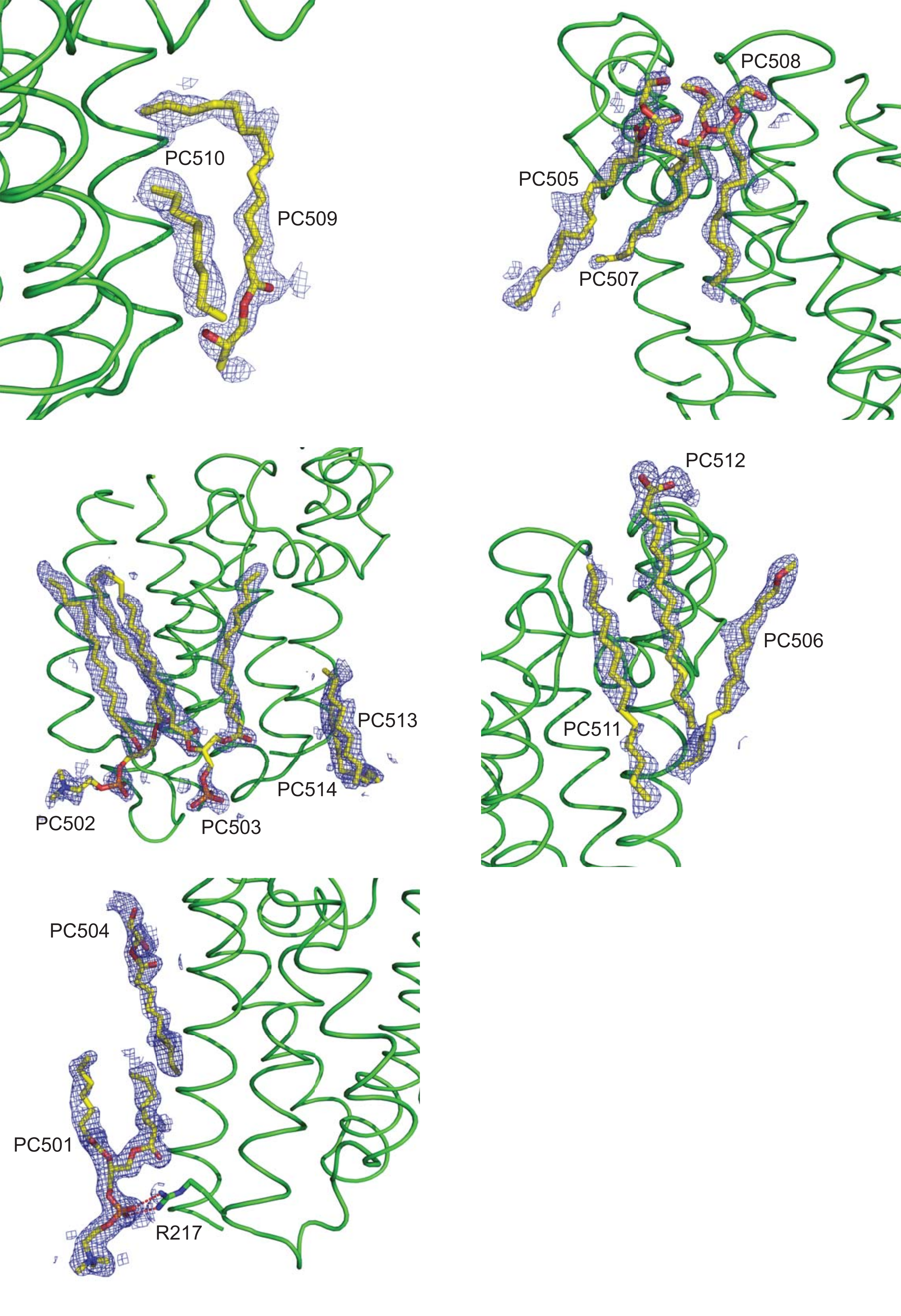

Supplementary figure 4

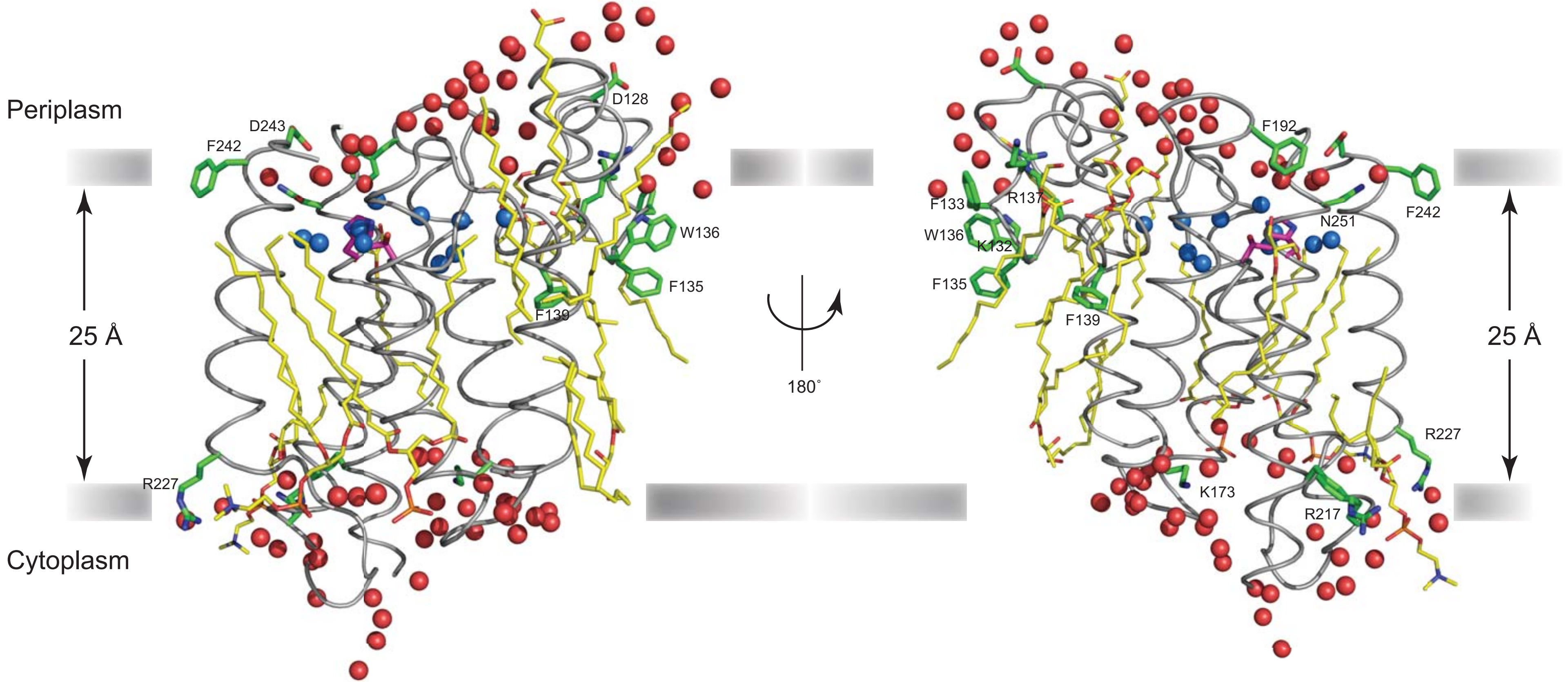

Supplementary figure 5

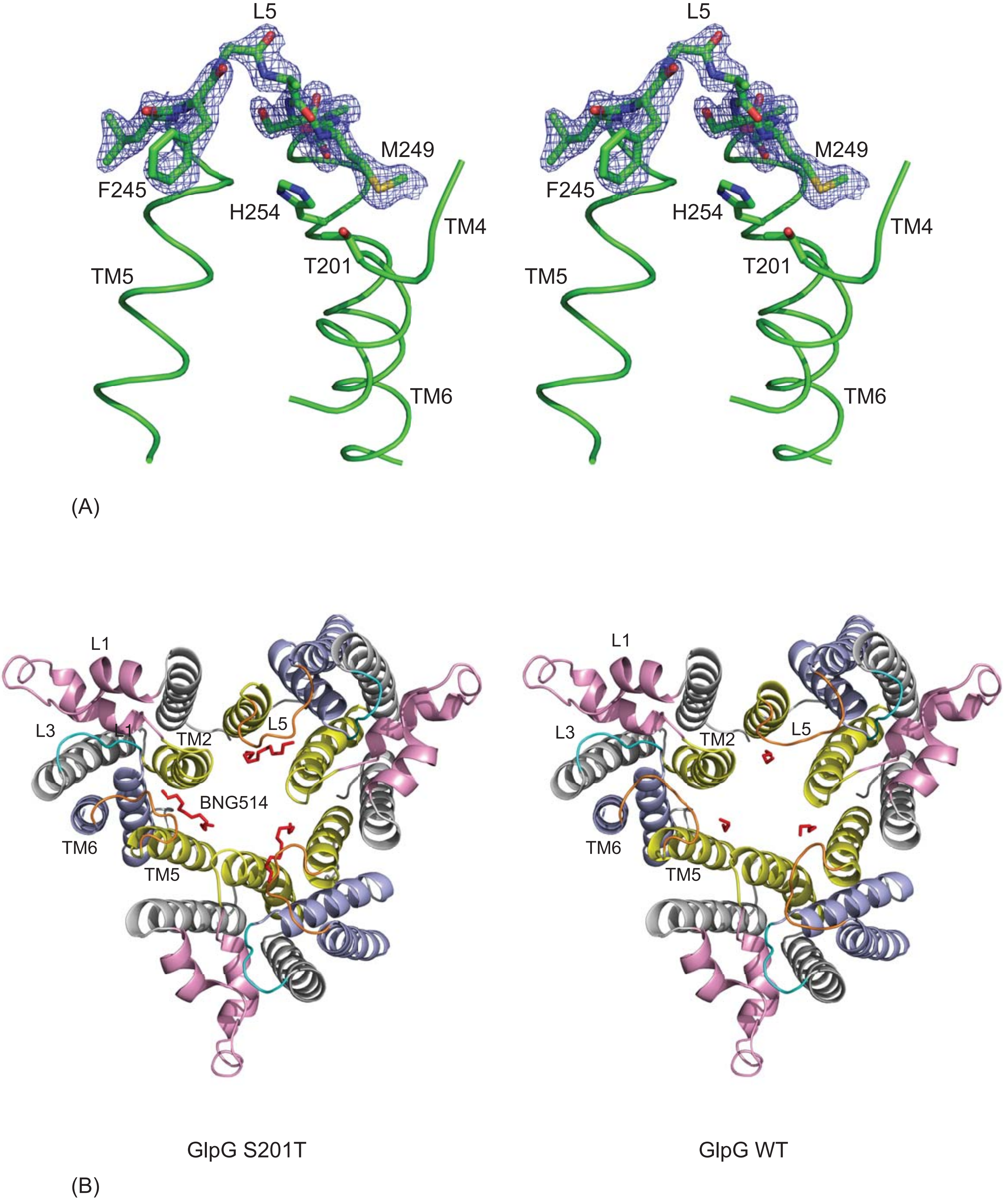

Supplementary figure 6
